# APEX2-Mediated Proximity Labeling of Wnt Receptor Interactors Upon Pathway Activation

**DOI:** 10.17912/micropub.biology.000817

**Published:** 2023-05-15

**Authors:** Ellen Youngsoo Rim, Roeland Nusse

**Affiliations:** 1 Department of Developmental Biology, Stanford University School of Medicine; 2 Howard Hughes Medical Institute

## Abstract

The Wnt signaling pathway regulates metazoan development, tissue homeostasis, and regeneration. Many outstanding questions in Wnt signal transduction revolve around the molecular events immediately following Wnt-receptor interactions. To identify binding partners of the Wnt receptor Frizzled 7 (Fzd7) upon pathway activation, we tagged Fzd7 with APEX2, an enzyme that allows biotinylation of proximal interactors with high temporal and spatial resolution. Upon confirming proper localization and signaling activity of APEX2-tagged Fzd7, we labeled proximal interactors of Fzd7 with or without Wnt3a stimulation. Mass spectrometry analysis of biotinylated interactors identified several known Wnt pathway proteins. Top interactors enriched upon Wnt treatment were involved in actin cytoskeleton regulation, vesicle trafficking, or phospholipid modification. Proteins enriched in the Wnt-activated Fzd7 interactome that are without established roles in Wnt signaling warrant further examination.

**Figure 1. APEX2-Mediated Proximity Labeling of Wnt Receptor Interactors Upon Pathway Activation:**
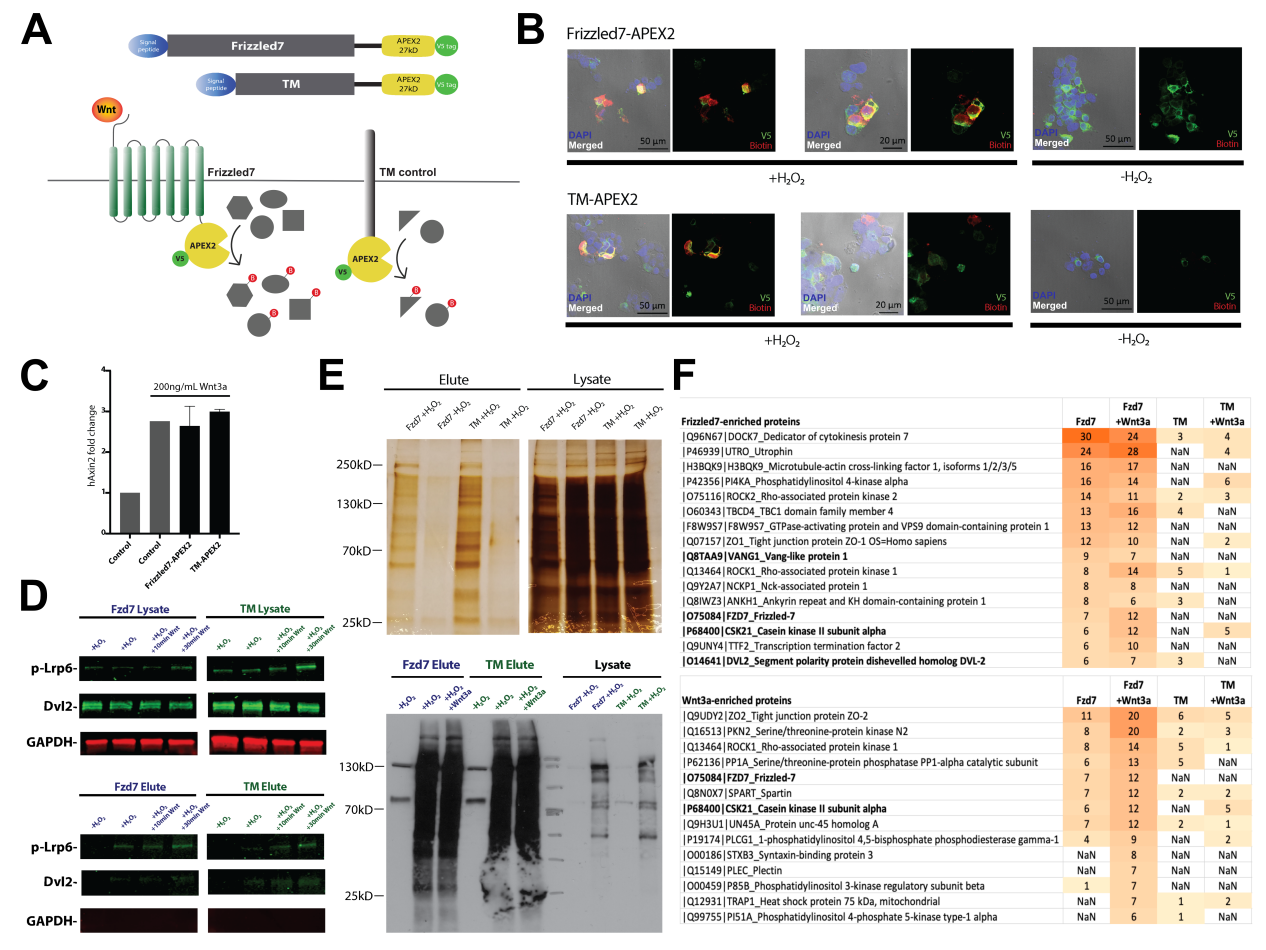
**(A) APEX2 tagging schematics.** Fzd7 and transmembrane (TM) localizing control were tagged with APEX2 at the cytoplasmic C-terminus along with a V5 epitope tag. Proximal interactors of Fzd-APEX2 and TM-APEX2 were biotin labeled with or without Wnt3a stimulation in HEK293T cells. **(B) Fzd7-APEX2 and TM-APEX2 localization and biotinylation activity.** Immunocytochemistry with anti-V5 (green) detected Fzd7-APEX2 and TM-APEX2 at the plasma membrane of HEK293T cells. Streptavidin-Cy5 (red) detected biotinylated proteins in cells treated with H2O2. **(C) Wnt signaling in cells expressing Fzd7-APEX2 and TM-APEX2.** qPCR confirmed transcription of Axin2 upon 2-hour stimulation with 200ng/mL Wnt3a in HEK293T cells transfected with Fzd7-APEX2 and TM-APEX2. **(D) Biotinylation of Wnt pathway components by Fzd7-APEX2** . Top: Western blot detected phosphorylated Lrp6 (p-Lrp6) in lysates treated with Wnt3a. Bottom: p-Lrp6 and Dvl2 were detected in the biotinylated elutes in a H2O2-dependent, but not Wnt3a- or Fzd7-dependent manner. The cytoplasmic control GAPDH was detected in lysates but not in elutes. **(E) Immunoprecipitation of biotinylated interactors of Fzd7-APEX2 and TM-APEX2.** Top: Silver staining of elutes and lysates. Elute fractions from cells treated with H2O2 confirmed immunoprecipitation of proteins biotinylated by Fzd7-APEX2 and TM-APEX2. Bottom: Western blot confirmed enrichment of biotinylated proteins upon Streptavidin-mediated immunoprecipitation of samples treated with H2O2. **(F) Mass spectrometry identification of Fzd7-APEX2 interactors.** Top: List of proteins enriched in Fzd7-APEX2 samples compared to TM-APEX2 samples. Proteins of interest that showed at least 3.5-fold enrichment in Fzd7-APEX2 samples over TM-APEX2 samples were ranked by spectral count. Each protein entry lists the Uniprot identifier, protein symbol, and protein full name. Bottom: List of proteins enriched in the Fzd7-APEX2 sample treated for 30min with 200ng/mL Wnt3a and depleted in TM-APEX2 samples. Proteins of interest that showed at least 70% enrichment upon Wnt3a treatment were ranked by spectral count. A full list of proteins identified through mass spectrometry analysis can be found in supplementary data.

## Description

Many outstanding questions in the Wnt field revolve around the proximal events in Wnt signal transduction (Rim et al. 2022) . What molecular events follow Wnt ligand binding to the co-receptors Lrp and Frizzled (Fzd) at the cell membrane, and how do the events lead to inhibition of β-catenin phosphorylation in the cytoplasm? Interaction of Fzd receptors with other Wnt pathway proteins such as Lrp6 and Dvl upon signaling initiation is well documented (Cong 2004; Tamai et al. 2004) . However, we do not understand how these interactions elicit downstream events and whether other molecular interactors of Fzd are involved in signal transduction. Here we designed an approach to probe interaction partners of Fzd7 using a biochemical labeling approach with high temporal and spatial resolution. APEX2 is an engineered ascorbate peroxidase that can capture the immediate biochemical environment of a protein of interest. Briefly, APEX2 catalyzes biotinylation of endogenous proteins that are up to several nanometers away upon addition of biotin phenol and hydrogen peroxide (Hung et al. 2016a). Importantly, APEX2 can carry out biotin labeling in live cells in less than a minute. Therefore, APEX2-mediated biotin labeling allows detection of transient or weak interactors which might escape detection in a conventional co-immunoprecipitation approach.

Fast kinetics and live cell labeling render APEX2 suitable for probing interactors of receptors whose signal transduction function depends on rapid changes in their protein interaction network. For instance, APEX2 has been utilized to identify novel interactors of a well-studied GPCR, the beta-2 adrenergic receptor (Lobingier et al. 2017) . This study highlighted that, given the high labeling promiscuity of APEX2, an appropriate spatial control tagged with APEX2 is essential in order to deconvolve authentic interactors from labeled bystanders. Other studies used APEX2 labeling to analyze interactors of Fzd9 complexed with its unconventional co-receptor, EGFR, or interactors of the Wnt receptor Lrp6 (Colozza et al. 2020; Grainger et al. 2016) . These experiments identified endocytic machinery and endosomal proteins as interactors of Wnt receptors. To gain insight into changes in Fzd7 behavior following Wnt pathway activation, with a spatial control to deconvolve the interactome, we tagged Fzd7 and transmembrane (TM) spatial control with APEX2. Fzd7 was selected since it initiates β-catenin-mediated Wnt signaling in key physiological contexts (Fernandez et al. 2014; King et al. 2012) .

**Fzd7-APEX2 and TM-APEX2 Localize to the Membrane**

Along with mouse Fzd7, the TM sequence of PDGF receptor was tagged with APEX2 as a spatial control that localizes to the plasma membrane (Rhee et al. 2013) . TM-APEX2 can be used to deconvolve authentic Fzd7 interactors from nonspecific bystanders. Both Fzd7 and TM constructs were tagged with APEX2 at the cytoplasmic C-terminus to capture their intracellular interactors ( Figure 1A ). Along with APEX2, V5 epitope tag was attached to the C-terminus for recombinant protein detection and a signal peptide was attached to the N-terminus for proper membrane sorting. For introduction of Fzd7-APEX2 and TM-APEX2 into cells, we tested transient overexpression and stable integration using the Sleeping Beauty transposon system. Since expression level and biotinylation activity were low with the integration approach, we moved forward with the overexpression approach. Expression patterns of Fzd7-APEX2 and TM-APEX2 were assessed through V5 tag staining of transfected HEK293T cells. High signal at the membrane and low signal in the cytoplasm indicated that most of Frd7-APEX2 and TM-APEX2 localized to the plasma membrane ( Figure 1B ).

**Fzd7-APEX2 and TM-APEX2 Exhibit Robust Biotinylation Activity**

Biotin labeling by Fzd7-APEX2 and TM-APEX2 was induced through addition of biotin phenol substrate followed by 1-minute incubation with hydrogen peroxide. We detected biotinylated products through immunocytochemistry and Western blotting with Streptavidin ( Figure 1B and Figure 1E). High levels of biotinylation were observed in cells transfected with Fzd7-APEX2 and TM-APEX2 and pulsed with hydrogen peroxide, but not in cells untreated with hydrogen peroxide. Bands at 75kD and 130kD in the negative control samples without hydrogen peroxide reflect proteins known to be endogenously biotinylated (Han et al. 2017) . Streptavidin-mediated immunoprecipitation enriched for biotinylated proteins in the samples, as shown by silver staining and Western blotting of the elute fractions ( Figure 1E ). After confirming the biotinylating activity of Fzd7-APEX2 and TM-APEX2 and immunoprecipitation of biotinylated targets, we proceeded to assess Wnt signaling in cells transfected with the APEX2-tagged proteins.

**Wnt Signaling Proceeds in Cells Expressing Fzd7-APEX2 and TM-APEX2**

HEK293T cells transfected with Fzd7-APEX2 and TM-APEX2 activated Wnt signaling in response to Wnt3a treatment, as shown by increased phosphorylation of Lrp6 ( Figure 1D ), a hallmark of signaling initiation, as well as downstream induction of target gene Axin2 ( Figure 1C ) (Davidson et al. 2005; Jho et al. 2002; Lustig et al. 2002) . Therefore, APEX2-tagged Fzd7 and TM do not interfere with Wnt signal transduction in these cells.

Biotinylation by Fzd7-APEX2 or TM-APEX2 was carried out with or with- out Wnt3a treatment of the cells. Biotinylated interactors were eluted then blotted for known endogenous Wnt pathway components ( Figure 1D ). Phosphorylated Lrp6 (p-Lrp6) and Dishevelled2 (Dvl2) were detected in the elutes in a hydrogen peroxide-dependent, but not Fzd7-specific manner ( Figure 1D, bottom ). Overall interaction of p-Lrp6 with Fzd7-APEX2 was slightly higher compared to that with TM-APEX2 ( Figure 1D, bottom ). However, there were no noticeable Wnt-induced interaction changes specific to Fzd7. Since a high-throughput, high-sensitivity analysis of the biotinylated interactome might yield genuine, Wnt-specific factors that associate with Fzd7-APEX2, mass spectrometry analysis followed.

**Mass Spectrometry Identifies Known Wnt Pathway Components and Novel Interactors**

Mass spectrometry was performed on four samples: Fzd7-APEX2 interactors with or without 30-minute treatment with 200ng/mL Wnt3a and TM-APEX2 interactors with or without the Wnt3a treatment. In each condition, 1000-1300 protein targets were identified and contaminants such as human keratins were excluded. Top table in Figure 1F lists proteins at least 3.5-fold enriched in the Fzd7 interactome but undetectable or depleted in the TM interactome. Bottom table in Figure 1F lists proteins preferentially labeled by Fzd 7-APEX2 and shows greater than 70% enrichment in the Wnt3a-treated sample versus the untreated sample. A full list of proteins identified through mass spectrometry can be found in the supplementary data.

Cytoplasmic signal transducers of β-catenin-dependent Wnt signaling such as Dvl2 and CK2 and a transmembrane protein that interacts with Fzd in β-catenin-independent Wnt signaling, Vang-like protein 1, were enriched in Fzd7-APEX2 samples ( Figure 1F, top ). Among the proteins that increased their interaction with Fzd7-APEX2 upon Wnt3a treatment were Fzd7 itself and the alpha subunit of CK2 ( Figure 1F, bottom ). It is possible that multiple molecules of Fzd aggregate or come in proximity upon pathway activation (Hirai et al. 2019) . CK2 is a kinase reported to be essential for Wnt signal transduction although it remains unclear which aspect of its phosphorylative activity is important (Gao & Wang 2006; Song et al. 2000) . Surprisingly missing from the list are the Lrp co-receptors, which are known to form a complex with Fzd upon Wnt binding (Tsutsumi et al. 2022) . Similarly, Axin is not present within the Fzd7- or Wnt activation-specific interactors we identified, although Axin is thought to be recruited to Lrp5/6 upon Wnt activation (Tamai et al. 2004) . One possibility is that Fzd7 interaction with these components is short-lived, with dissociation complete within 30-minutes. Another possibility is that Fzd7 and Lrp5/6 or Axin do not come in close proximity in this context, perhaps because the behavior of Fzd7-APEX2 does not faithfully reflect that of endogenous Fzd7 or only reflect that in the β-catenin-independent pathway.

Many of the proteins enriched in the Wnt-activated conditions have less well-established connections to Wnt signaling and could be examined further. Some are interactors of Rho GTPases involved in actin cytoskeleton regulation. PKN2 is a kinase required in mouse development with roles in early morphogenesis and cell migration (Quétier et al. 2016). It is implicated in the planar cell polarity pathway, a β-catenin-independent branch of Wnt signaling that mediates tissue polarity establishment. Rho-kinase 1 (ROCK1), another protein with a role in the planar cell polarity pathway, mediates phosphorylation of actin cytoskeleton regulators. Another protein enriched in the interactome of Wnt-activated Fzd7 is PIP5K1. PIP5K1 is a lipid kinase that interacts with Dvl upon Wnt pathway activation, which may play a role in signal transduction (Hu et al. 2015; Mahoney et al. 2022) . PIP5K1 generates the phospholipid species PtdIns4,5P2, which might enhance Wnt signal transduction through either regulation of Lrp6 phosphorylation or receptor complex formation (Hu et al. 2015; Kim et al. 2013) . These examples present interesting targets for follow-up studies. Together, our data demonstrate that rapid labeling in live cells with APEX2 can identify interaction networks of Wnt signal transducers and changes thereof, and provide new targets in the Fzd7-interactome upon pathway activation.

## Methods

**Cell culture and reagents**

HEK293T cells were cultured and stimulated with Wnt3a as in (Rim et al. 2020) . Briefly, HEK293T cells were maintained in DMEM with GlutaMAX supplemented with 10% fetal bovine serum, 100 U/ml penicillin, and 100 μg/ml streptomycin. Career-free recombinant mouse Wnt3a was reconstituted in 0.1% bovine serum albumin in PBS and added to culture at specified concentrations.

**DNA constructs**

Mouse Frizzled7 coding sequence with C-terminal APEX2 and V5 tag was cloned into vectors for either transient expression or Sleeping Beauty transposon-mediated integration into the genome. For transient expression, pCS2+ vector with a CMV promoter was used (Plasmid #27242 from Addgene (Tamai et al. 2000) ). For Sleeping Beauty-mediated integration, modified version of pT2/SVNeo (Plasmid #26553 from Addgene (Cui et al. 2002) ) with the human promoter EF1α and mammalian bGH polyA signal was used. For the spatial control, transmembrane domain of PDGF receptor from pCAG HRP-TM (Plasmid #44441 from Addgene (Rhee et al. 2013) ) was tagged with C-terminal APEX2 and V5 tag and cloned into the same transient expression or integration vectors.

**Reverse transcription and quantitative real-time PCR**

To activate Wnt signaling, cells were stimulated for 2 hours with 200ng/mL Wnt3a in medium. Total RNA was prepared using the RNeasy mini kit and reverse transcribed using random primers. Gene expression was assayed by real-time PCR using TaqMan Gene Expression Assays on Applied Biosystems StepOne Plus real-time PCR system. All PCRs were carried out in triplicates with glyceraldehyde-3-phosphate dehydrogenase (GAPDH) as a reference gene. The following TaqMan probes were used: human Axin2 (Hs00610344) and human GAPDH (4326317E).

**APEX2 biotinylation**

APEX2 labeling was carried out following the protocol published by Hung and colleagues (Hung et al. 2016b). Cells were pulsed with 500μM biotin tyramide in pre-warmed culture medium for 30 min at 37◦C. To activate Wnt signaling, cells were stimulated with 200ng/mL Wnt3a in medium at the same time. To induce labeling, fresh stock of 100mM H2O2 in PBS was added to medium to 1mM final concentration. After 1 min incubation at room temperature, cells were washed 3x in quencher solution [10mM sodium azide, 10mM sodium ascorbate, and 5mM Trolox in PBS] to remove excess biotin phenol probe. Cells were then lysed in RIPA buffer [150 mM NaCl, 1% IGEPAL CA-630, 0.5% of sodium deoxycholate, 0.1% sodium dodecylsulfate (SDS), and 50 mM Tris] supplemented with protease inhibitor cocktail and 1mM PMSF. Cell lysates were clarified by centrifugation at 16,000g for 10 min at 4◦C. Total protein concentration of the lysates was measured using Pierce BCA Assay.

**Immunocytochemistry and microscopy**

Biotinylation was carried out as described above, with H2O2 omitted in negative control samples. Cells were fixed with 4% paraformaldehyde in PBS for 8 min at room temperature, washed 3× with staining buffer [0.1% Tween-20, 1% BSA, 0.05% sodium azide in PBS], and blocked for 1 hour in 2% FBS in staining buffer. Cells were incubated overnight at 4◦C in primary antibody in staining buffer. Primary stain was followed by 3× washes with staining buffer then 1 hour room temperature incubation with secondary antibody or fluophore-conjugated Streptavidin in staining buffer. Cells were washed 3× and mounted in Prolong Gold Antifade Mountant with DAPI, dried overnight, and imaged.

**SDS gel silver stain**

Proteins in prepared elutes and lysates were resolved via SDS-PAGE in 10% Mini- PROTEAN Tris-Glycine gels. SDS gel was incubated in fixing solution [30% ethanol, 10% acetic acid] for 15 min then in new fixing solution for another 15 min. Silver staining was carried out using Pierce Silver Stain Kit according to the manufacturer’s protocol. Gel was kept in stain working solution for 30 min then developed for 30 sec to 1 min. After a 10 min wash in 5% acetic acid solution, stained gel was imaged on Bio-Rad ChemiDoc.

**Immunoprecipitation of biotinylated proteins and Western blots**

Biotinylated proteins were immunoprecipitated using Streptavidin magnetic beads. 500μg magnetic beads were incubated with up to 100μg of lysate in 300μL RIPA lysis buffer rotating overnight at 4◦C. Beads were washed 2× with RIPA buffer, 1× with 2M urea in 10mM Tris-HCl, then 2× with RIPA buffer. Elution buffer was prepared by diluting 6x Laemmli sample buffer [300mM Tris-HCl, 10% SDS, 30% Glycerol, 500mM DTT, 150μM Bromophenol blue] to 1x with RIPA buffer and supplementing with 2mM Biotin and 20mM DTT. Biotinylated proteins were eluted in by heating in 60μL elution buffer for 5 min at 95◦C. Proteins in prepared elutes and lysates were resolved via SDS-PAGE. Following antibodies were used for Western blots: Streptavidin-HRP (0.2μg/mL), phospho-Lrp6 (1:1000), Dvl2 (1:1000), GAPDH (1:1000).

**Mass spectrometry**

Mass spectrometry analysis was performed by Stanford University Mass Spectrometry. Streptavidin magnetic beads were subjected to on-bead trypsin digestion of immunoprecipitated interactors. Purified peptides were loaded onto a NanoLC C18 analytical column then subjected to ionization and sequencing on Orbitrap mass analyzer. Peptide sequences were searched against HEK293T proteome to generate a ranked list of matched proteins. A full list of proteins identified through mass spectrometry can be found in supplementary data.

## Reagents

**Table:** 

DMEM with GlutaMAX	Thermo Fisher 10569010
Fetal bovine serum	Omega Scientific FB-01
Penicillin-Streptomycin	Thermo Fisher 15140122
Recombinant Mouse Wnt-3a Protein	R&D 1324-WN/CF
RNeasy Mini Kit	Qiagen 74104
High Capacity cDNA Reverse Transcription kit	Applied Biosystems 4368814
Human Axin2 TaqMan probe	Thermo Fisher 4331182 (Hs00610344_m1)
Human GAPDH TaqMan probe	Thermo Fisher 4326317E
Biotin tyramide	Iris Biotech LS-3500
Protease inhibitor cocktail	Abcam ab65621
Pierce BCA Protein Assay Kit	Thermo Fisher 23225
Prolong Gold Antifade Mountant with DAPI	Thermo Fisher P36931
V5 Tag Monoclonal Antibody	Thermo Fisher R960-25
Donkey anti-mouse IgG secondary antibody, Alexa Fluor 488	Invitrogen A-21202
Streptavidin-Cy5	Invitrogen 438316
10% Mini-PROTEAN TGX Precast Protein Gels	Bio-Rad 4561033
Pierce Silver Stain Kit	Thermo Fisher 24612
Pierce Streptavidin magnetic beads	Thermo Fisher 88816
Streptavidin, horseradish peroxidase conjugate	Thermo Fisher S911
Phospho-LRP6 (Ser1490) Antibody	Cell Signaling Technology 2568
Dvl2 (30D2) Antibody	Cell Signaling Technology 3224
GAPDH Antibody	Proteintech 60004-1

## Extended Data

Description: Spectral counts of biotin labeled interactors of Frizzled7-APEX2 and TM-APEX2 with or without Wnt3a. Resource Type: Dataset. DOI: 10.22002/s4a2j-bmg04
